# The Distribution of the Hospital Sunday Fund

**Published:** 1897-08-14

**Authors:** 


Vol. XXII.—No. 568.] [Aug. 14, 1897.

### The Distribution of the Hospital Sunday Fund.

As we said last week, it is satisfactory to find that the money already available for distribution by the Metropolitan Hospital Sunday Fund is only £2,000 less than the whole sum distributed last year. This proves that the Prince of Wales's Fund has not interfered with the collections in the churches, a considerable number of which are infinitely larger than they have been in any previous year. One of the most zealous and staunch friends of the Hospital Sunday Fund is the Chief Rabbi, the Rev. Dr. Adler. This year the Jewish congregations have made a special effort on behalf of the hospitals, with the result that the sum received from this source shows an increase of at least 30 per cent. over any previous year. Canon Fleming and the Rev. Mr. Ridgway again head the churches' collections so far as the sums raised are concerned, but upwards of one hundred other clergymen have carefuly studied and adopted the methods pursued by these gentlemen, with the result that they have increased the contributions of their congregations to a gratifying extent. All honour to the clergy and ministers of religion who especially exerted themselves on behalf of the hospitals, to which the poor of their parishes are so greatly indebted for succour in sickness and for help and sustenance in the days of accident and emergency.

We have often said, and we repeat here, that if it were possible to make every clergyman and minister of religion realise that to help the hospitals on Hospital Sunday, and to secure the largest possible collections on that day, is to ensure for every other object for which they may appeal during the year a larger contribution instead of a smaller one, as so many of them inaccurately contend, then the sum raised on Hospital Sunday in London would never be less than £100,000. It would then be worthy of the religious sentiment of the metropolis of the Empire. Owing to various causes an unusually large number of places of worship have not yet paid in the collections for the hospitals during the current year. The accounts of the Hospital Sunday Fund do not close until October 31st, by which date there is every reason to expect that the additional sums to be received will bring up the collections to an amount at least equal to that received during 1896, if the actual total is not even greater in 1897 than in the previous year. It is most satisfactory that the carping criticisms of the few thankless persons who never tire of abusing every fund promoted to help the hospitals, and especially the Hospital Sunday Fund, are thus shown to be unworthy of attention, for they seldom or never take the trouble to ascertain the facts before giving utterance to their ill-founded and irrelevant criticisms.

For twenty-five years the public have nobly responded to the earnest appeal made by the ministers of religion in nearly two thousand metropolitan places of worship on Hospital Sunday. During the whole of that time, under the zealous guidance of Sir Sydney Waterlow, the Distribution Committee of the Hospital Sunday Fund have laboured assiduously to distribute the moneys placed at their disposal with impartiality and justice. The work of distribution is no easy task. It entails a vast amount of labour and the sacrifice of much time on the part of the members of the Distribution Committee. As a preliminary to its meetings, nearly four months are devoted to a careful examination of the accounts of nearly two hundred institutions, which apply for grants. When the analyses of these accounts are ready the Distribution Committee commences to sit. They then go through the accounts of the expenditure and work done by each institution, and pay particular attention to the quality of the management, the economy of the administration, and the merits and pecuniary needs of each of the charities concerned. Each year it has been found desirable to hold a conference with the managing committees of certain institutions before deciding if any and what grant shall be made in these cases. During 1897 thirty-eight institutions were invited to send representatives to a conference of this kind. Thirty deputations were received by the Committee, and these conferences were found to be both useful and mutually instructive. In some cases the objections were removed or modified by the explanations given, and in others the result of the conference was to confirm the impressions the Committee had derived from the material at their disposal. When it is remembered that the average time entailed by each conference was one hour, it will be seen that the number of meetings which the Distribution Committee had to hold was considerable, and that the work done has probably been more onerous this year than ever before. The results, as testified by the list of grants made, which will be found in another column of the present issue, cannot fail to excite special interest, and to command the approval of the public. There is no more difficult or thankless task than the distribution of money, and the fact that the twenty-five years during which the Committee has sat have produced so little criticism, is a testimony to the excellence, thoroughness, and impartiality with which they have discharged their onerous duties.

How little substance there is in the criticisms to which the Distribution Committee is subject is proved by a recent instance. The *Medical Press* in its last issue asks, "Does the Hospital Sunday Fund make any attempt whatever to regulate its grants in proportion to the wise and economical expenditure of individual charities? On the contrary, its chief condition is the relation of administration to maintenance, a fallacious condition that simply supplies a direct incentive to reckless expenditure." We have already shown in general terms that this statement is inaccurate and untrue in substance and in fact. No unprejudiced writer with any adequate sense of responsibility would have ventured to make it, because he would have enquired into the facts, and so have found that his impressions were incorrect. The statement of the *Medical Press* as it stands can be tested in various ways by a reference to the awards, which are given on page 344 of our present issue. Let us take one instance. The Chelsea Hospital for Women, and the Hospital for Women, Soho Square, both receive the same grant, £306 5s. These hospitals contain about the same number of beds, the average number of occupied beds being nine less in the latter than in the former hospital, whilst the latter treated double the number of out-patients. The percentage of management to maintenance was 14½ per cent. in the case of the Chelsea Hospital for Women, and 12·8 per cent. in the case of the Hospital for Women, Soho Square. It follows that if the statement made by the *Medical Press* were true, the Hospital for Women would have received a larger grant than the Chelsea Hospital, but a consideration of the merits and needs of the respective institutions has resulted in the awards being identical in both instances.

There is, however, one point in connection with the distribution of the Sunday Fund which we should like to have thoroughly discussed. The laws of the constitution provide that the payments made by or on behalf of patients shall be dealt with in each case as the Committee of Distribution may see fit. It was pointed out at the meeting at the Mansion House on the 5th inst., by Sir Sydney Waterlow, that the reason why one of the best-managed hospitals in London—the National Hospital for the Paralysed and Epileptic—received a less grant than Charing Cross Hospital and the Royal Free Hospital, although the number of beds in each of these institutions is nearly the same, was, that the first of the three hospitals received £2,000 a year from payments made by patients. Sir Sydney Waterlow further stated that the Distribution Committee did not deduct all the contributions made by patients from the basis of award, but we are of opinion that, so far as in-patients are concerned at any rate, none of such payments should be deducted. Our view is based on the feeling that there is great danger that such deductions might create the impression that all attempts to make the patients thrifty and to encourage them in habits of self-respect were opposed to the principles upon which the funds of the Hospital Sunday Fund were distributed. Such an impression would be a grave calamity, seeing that one of the most hopeful directions to which we look for necessary reform in our modern hospital system is a plan whereby each hospital shall be able to classify the applicants for relief according to their needs and social position. This would secure that, whilst all patients entitled to treatment by reason of their physical condition are admitted to relief, no patient will receive free relief at a hospital who is not in want of it and entitled to it.

We are confident that it is the duty of the Distribution Committee, and of everybody who has the welfare of the community as well as that of the hospitals at heart—to say nothing of the claims of the general practitioners of medicine, probably the most urgent of all—to use the whole of their influence in the promotion of thrift, the building up of self-respect amongst the classes who frequent the hospitals, and to afford them full facilities to pay according to their means for the medical relief which they receive. Whereas it is dangerous, because it is apt to set back the clock, to impose an arbitrary fee upon out-patients at a hospital, it is helpful and politic to charge the out-patients a definite fee (with certain exceptions to prevent the exclusion of the deserving poor), such sum representing either the ascertained cost of the medicine, or an ascertained portion of the actual expenditure entailed by the patient's treatment at the particular institution from which relief is sought.

The confidence and authority which attaches to the work of the Distribution Committee of the Hospital Sunday Fund leads us to make a suggestion for Sir Sydney Waterlow's consideration. Will he not consent to prepare and read a paper in the month of October, explaining the whole system upon which the Hospital Sunday Fund is distributed? He would thus raise a discussion which might include the points we have just raised, and others which are of absorbing interest and importance to the whole community at the present time.

